# A dosimetry study precisely outlining the heart substructure of left breast cancer patients using intensity‐modulated radiation therapy

**DOI:** 10.1120/jacmp.v15i5.4624

**Published:** 2014-09-08

**Authors:** Ling‐li Fan, Yang‐kun Luo, Jing‐hui Xu, Ling He, Jie Wang, Xiao‐bo Du

**Affiliations:** ^1^ Department of Oncology Mianyang Central Hospital Mianyang Sichuan China; ^2^ Guangxi Medical University Nanning Guangxi China; ^3^ Department of Radiation Oncology Cancer Hospital of Sichuan Province Chengdu Sichuan China; ^4^ Sichuan Province Center for Disease Control Chengdu Sichuan China

**Keywords:** CTA, breast cancer, IMRT, cardiac substructures, dosimetry

## Abstract

The purpose of this study was to evaluate the feasibility of delineating the substructure of the heart by using 64‐slice spiral CT coronary angiography (CTA) in breast cancer patients who underwent left breast‐conserving surgery, and to compare the dosimetric differences between the targets and organs at risk in the prone and supine positions in intensity‐modulated radiation therapy (IMRT) planning. From January to December 2011, ten patients who underwent left breast‐conserving surgery were enrolled in this study. CTA was performed in both the supine and prone positions during the simulation, and conventional scanning without CTA was performed at the same time. Image registration was performed for paired image series using a commercially available planning system. In a conventional image series, the clinical target volume (CTV) of the whole breast, planning target volume (PTV), bilateral lungs (L‐Lung, R‐Lung), spinal cord, contralateral breast (R‐Breast), and heart were delineated. In the CTA image series, the left ventricular (LV) and left anterior descending coronary arteries (LAD) and the planning risk volume (LAD‐PRV) of the LAD (LAD with a 1 cm margin) were outlined. For each patient, two separate IMRT plans were developed for the supine and prone positions. A total of 20 plans were generated. The following indicators were compared: Dmean and D95 for the PTV; Dmean, V5, and V20 for the left lung; Dmean, V10, V20, V25, V30, and V40 for the heart and its substructures (LAD‐PRV, LV); Dmean and V5 for the right lung; and Dmax and Dmean for the right breast. Using CTA to delineate the substructures of the heart is simple and straightforward. Plans for both the prone and supine positions reached the prescribed dose for the PTV without significant differences. Dose distributions were acceptable for both the prone and supine positions. However, the LAD‐PRV, LV, heart, and L‐Lung received smaller doses in the prone position plans than in the supine position plans. The Dmean values reduced by 445.83cGy(p=0.043),575.00cGy(p=0.003),402.00cGy(p=0.039), and 553.33cGy(p=0.004) in the LAD‐PRV, LV, heart, and L‐Lung. In addition, the V25 lessened 12.54%(p=0.042) and 8.70%(p=0.019) in the LV and heart, while the V20 was decreased 8.57%(p=0.042),15.21%(p=0.026),12.59%(p=0.011), and 10.62%(p=0.006) in the LAD‐PRV, LV, heart, and L‐Lung, respectively. Similarly, the V10 and V30 were reduced by 28.31% (p=0.029) and 5.54%(p=0.034) in the heart, while the V5 was cut back 27.86%(p=0.031) in the L‐Lung. For most Asian women with average‐sized breasts after breast conserving treatment (BCT), prone positioning during IMRT radiation will reduce the dose to the ipsilateral lung, heart, and substructures of the heart, which may reduce the incidence of cardiovascular events after radiotherapy more than radiation therapy performed in a supine position. Using CTA to delineate the substructures of the heart is easy and intuitive. It is cost‐effective and highly recommended for breast cancer IMRT. However, the dose‐volume limits of the heart substructures remain to be determined.

PACS number: 87.55.dk

## I. INTRODUCTION

Breast cancer is one of the most common malignancies in women.^(1)^ With the improvement of diagnostic techniques, an increasing number of patients with breast cancer are newly diagnosed each year. Long‐term survival of patients with early breast cancer can be obtained through multi‐disciplinary treatments including surgery, chemotherapy, radiotherapy, endocrine therapy, and molecular targeted therapy. Cardiovascular events after radiotherapy have become the major cause of non‐breast cancer death in these patients. The cardiac toxicity of radiotherapy is thus an important issue that needs to be extensively studied. Most studies found that the cardiac toxicity of radiotherapy is closely related to damage to key substructures of the heart, including the left anterior descending artery (LAD) and left ventricular artery (LV), but especially the LAD.^2^, ^3^, ^4^, ^5^ Various approaches have been attempted to alleviate heart damage caused by radiation. The existing methods include the use of new radiotherapy techniques, such as intensity‐modulated radiation therapy (IMRT), and changes in treatment positioning, such as using the prone position with the purpose of protecting the LAD.

The development of IMRT of the radiation beam profile has led to a major advance in the treatment of breast cancer with respect to lowering the risks of both acute and late toxic effects associated with radiation therapy. The presence of moist desquamation did significantly correlate with pain or a reduced quality of life in breast cancer patients who received radiotherapy; a multivariate analysis found that the use of breast IMRT is significantly associated with a decreased risk of moist desquamation.^(6)^ Compared to the groups where a standard wedge compensator was used, significantly fewer patients in the IMRT group developed palpable induration as assessed clinically at the center of the breast, pectoral fold, inframammary fold, and at the boost site.^(7)^

The supine position has been conventionally used for radiotherapy after breast‐conserving surgery.^8^, ^9^, ^10^, ^11^, ^12^ This technique has many advantages compared to prone positioning, including patient comfort, skin tag visibility, good immobilization, and reproducibility. In addition, the axillary lymph nodes can be better delineated and included in the treatment field than in the prone position. Patients with advanced stages of disease and those at a high risk for lymph node recurrence are thus recommended to be treated in a supine position. Nevertheless, the supine position possesses some disadvantages.^(3)^ For patients with large, sagging breasts, the supine position will cause more skin overlapping and increases the skin dose, which is unfavorable for cosmetic requirements. In addition, the uneven dose distribution will lead to long‐term edema and fibrosis in high‐dose regions. The adjacent normal tissues, including the ribs, heart, and lung, might receive a higher dose in the supine position than in the prone position because the breast tissue above these regions becomes thinner in this position. The supine position is, thus, not recommended for patients with large breasts, and prone positioning is a better choice for this group. Recently, there have been an increasing number of studies on treating patients with breast radiation in the prone position.^10^, ^11^, ^12^, ^13^, ^14^, ^15^ By pulling the breast away from the chest wall, this position can reduce the dose to the lung, heart, and contralateral breast. The breast becomes longer and narrower, which provides better dose distribution and uniformity.^9^, ^11^, ^15^, ^16^, ^17^, ^18^

To protect the LAD, precise delineation is required. The structure of the LAD was previously delineated based on the anatomic structure of the heart, which may have great inter‐ and intra‐observer bias.^15^, ^19^ Recently, single photon emission computed tomography (SPECT) and positron emission tomography (PET‐CT) were also used to identify the LAD.^(20)^ However, SPECT is better suited to myocardial imaging than to cardiac vessel imaging; both modalities, but especially PET‐CT, are costly procedures.

Therefore, an easy, precise, and inexpensive method to help delineate the LAD needs to be developed. With the advances in CT imaging technologies, including the introduction of ECG‐gated multidetector systems, heart and coronary imaging has become possible. Compared with invasive angiography, a noninvasive approach promotes the rapid growth of CT‐based heart imaging. CTA is commonly used in these noninvasive approaches. The visualization of the coronary anatomy and left ventricular function offered by CTA can be used to evaluate patients with suspected or diagnosed coronary heart disease. Noninvasive CTA technology requires a system with high spatial resolution that can track free movement within 20 s during a single breath‐hold; 64‐slice CTA has the full potential to accomplish this.

In this study, we used 64‐slice spiral CTA to visualize the substructure of the heart and to generate IMRT plans in the prone and supine position. We sought to make dosimetric comparisons of the target and normal tissues in the prone and supine positions.

## II. MATERIALS AND METHODS

### A. General clinical data

From January to December 2011, a total of ten patients who received BCT at Sichuan Cancer Hospital were enrolled in this study. The patients were all between 30 and 48 years of age, with a median age of 38 years; their breast sizes ranged from 183 to 401 cm^3^, with a median of 263 cm^3^. None had coronary heart disease, myocarditis, congestive heart failure, or any other history of heart disease before radiotherapy. The patients had normal findings on electrocardiography, cardiac enzyme tests, c troponin T (cTnT), and color Doppler energy (CDE) inspections. All patients provided written, informed consent.

### B. Patient immobilization and CT simulation

Thermal plastic masks were made for both supine and prone positioning. The patient was immobilized in the supine position with a supine mask, whereas in the prone position, the patient was immobilized with both a thermal plastic mask and a Biolnix RT‐6025 (Ruili Company, China) chest prone system. Before the simulation, the inferior–superior and left‐right borders of the breast were labeled with radio‐opaque lead wires; surgical scars were labeled, as well. The reference points were identified as the intersections between the body midline, the level of the nipples, and the middle axillary line.

CT simulations were performed on a GE VCT LightSpeed 64‐slice (GE Healthcare Technologies, Waukesha, WI) scanner with a single axial rotation in service mode. CTA data were analyzed on a computer workstation (Advantage Workstation, AW4.4, GE Medical Systems). After an injection of 20 mL of a high concentration of a contrast agent (370 mg I per mL) at a 5 mL/s flow rate through the cubital vein, a monitoring scan was performed. A region of interest for density tracking was plotted in the ascending aorta area at the level of the carina. The time‐density curve was obtained and the peak time was chosen to initiate the imaging sequence acquisition. The scanning level was from above the carina to the diaphragmatic surface of the heart. The main scanning parameters were as follows: scanning thickness, 0.625 mm; tube voltage, 120–140 kV; tube current, 350–380 mA; scanning pitch, 0.18; X‐ray tube rotation speed, 0.4 s; and scanning time, 5–7 s. A conventional scan of the breast was acquired immediately after the special sequence scanning. The range was from the submandibular region to 5 cm inferior to the lower edge of the contralateral breast. The scanning parameters were as follows: slice thickness, 2.5 mm; tube voltage, 120–140 kV; tube current, 350–380 mA; scanning pitch, 0.913; X‐ray tube rotation speed, 0.4 s; and scanning time, 5–7 s.

Image registration was performed using the radiation treatment planning system Oncentra MasterPlan version 4.1 (Nucletron, Stockholm, Sweden). The angiographic image series were reformatted to 2.5 mm in thickness and fused to the conventional image series.

The scanning was performed in the prone position on Day One and in the supine position on Day Two for each patient. The patients were advised to drink plenty of water after being scanned with an intravenous contrast agent.

### C. Radiotherapy target volume and normal tissue delineation

The target volume and organs at risk were delineated on conventional CT images using the MasterPlan radiation treatment planning system with a window width of 400 HU and a window level of 20 HU. The breast was delineated following the Radiation Therapy Oncology Group (RTOG) guidelines.^(21)^ The upper, inferior, and anterior margin of the target were placed at the sternoclavicular joint, clinical glands, and 0.5 cm under the skin, respectively. The posterior margin of the target was placed at the muscles and ribs, and the midline was marked. The clinical target volume (CTV) included the ipsilateral breast and the pectoralis major fascia. The skin, pectoralis muscles, ribs, and intercostal muscles were excluded.

The planning target volume (PTV) included an expansion of 0.6 to 0.9 cm beyond the CTV in the prone position in the direction of the head and feet, and a similar expansion of 1.0 to 1.5 cm in the supine position. Similarly, the PTV included an expansion of 0.3 to 0.5 cm beyond the CTV in the prone position in the direction of the sternum, armpits, and lung, and a similar expansion of 0.5 to 1.0 cm in the supine position. The skin direction was not extended (subcutaneous limit, 0.5 cm).

The outline of the heart, contralateral breast, lung, and spinal cord were sketched on a conventional sequence for the positioning of the CT image. The heart volume was delineated to include the heart and the ascending aorta and excluding the inferior vena cave. The lung was delineated automatically with the MasterPlan planning system. The contralateral breast was delineated by the breast parenchyma visible on the CT images. The LV regional wall and LAD were delineated on the special positioning CT image sequences. The LAD stems from the left main coronary artery and tracks along the anterior interventricular sulcus. As described in detail in the studies by Yu and Feng et al.,^20^, ^22^ the LV and LAD volumes were determined with the agreement of a senior physician from the diagnostic radiology department. The LAD‐PRV was defined as the LAD with a 1 cm margin.

**Figure 1:**
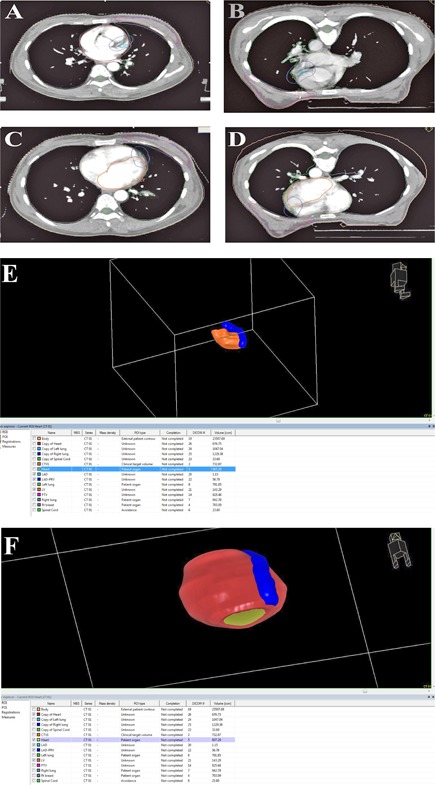
Sketch of the target and normal tissues in the same patient in different postures: (a) and (c) supine position; (b) and (d) prone position; and colored 3D reconstruction of the subcardinal organs, together with the heart (e) and (f). Blue=LAD‐PRV; orange=LV; chartreuse=heart.

The outline of the LV regional wall was clearly shown in special positioning CT images. With CTA imaging, the whole LAD can be easily distinguished and delineated in the anterior interventricular sulcus (Fig. 1).

### D. Treatment design

A dose of 5000 cGy in 25 fractions over five weeks was prescribed at the 100% isodose line. The entire PTV was encompassed by the 95% isodose line; the maximum dose of the PTV must be less than 110% of the prescription dose. The ipsilateral lung dose‐volume histogram (DVH) goals were V20<25% and mean dose <15Gy. The whole lung DVH goal was V20<20%. The heart DVH goals were V30<10% and V40<5%. The contralateral breast DVH goals were mean dose <5Gy and maximum dose <10Gy.^(23)^ The plans were evaluated and approved by two senior physicians in the Department of Radiotherapy.

IMRT planning was based on a 23EX linear accelerator (8 MV, 120 leaves) (Varian Medical Systems, Palo Alto, CA). The scale of our Linac is IEC Varian. For a tangential breast technique, four to six fields were used, and the field gantry angles were 280°, 250°, 220°, 180°, and 40°; and 300°, 320°, 100°, and 125° for the prone and supine positions, respectively, in left breast cancer patients.

### E. Plan comparisons

All radiation treatment planning designs have been optimized by direct machine parameter optimization. IMRT plans of each patient were created for the supine and prone positions, resulting in a total of 20 plans. Dose‐volume histograms (DVH) for the treatment volume and the organs at risk were generated by the treatment planning system and the following factors were compared between the supine and prone plans for each patient. The factors included Dmean and D95 of PTV; Dmean, V5, and V20 of the ipsilateral lung (L‐Lung); Dmean, V10, V20, V25, V30, and V40 of the heart and its substructures; Dmean and V5 of the contralateral lung; and Dmax and Dmean of the contralateral breast.

### F. Statistical methods

Data are expressed as the mean±standard deviation (±SD. A Statistical Package for the Social Sciences (version 13.0, SPSS Inc., Chicago, IL) was used for all statistical analyses. A paired Student's *t*‐test was used to compare different groups. P‐values <0.05 were considered statistically significant.

## III. RESULTS

**A. Dosimetric difference between prone and supine position plans**

Both plans met the dose requirements for the treatment volume and were approved by two senior physicians. All plans had a proper distribution (2, 3) and the difference was not statistically significant (Table 1).

**Figure 2:**
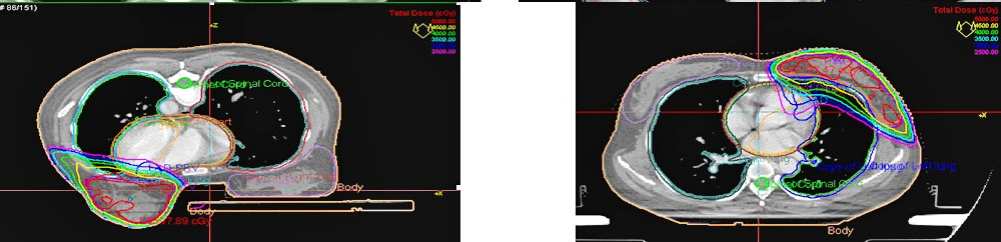
The dose distribution of the same patient in different body postures.

**Figure 3:**
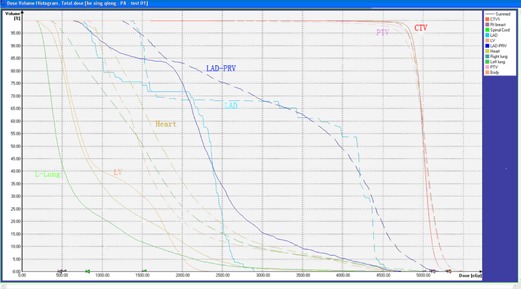
The DVH view of the same patient in different postures. The solid line represents the prone position, the dotted line represents the supine position.

**Table 1:** The target mean doses in the prone and supine positions (n=10)

*Target*	*Dose (cGy)*	*Prone Plan*	*Supine Plan*	*p‐value*
PTV	Dmean	5062.83±36.18	5041±77.18	0.526
	D95	4807.99±46.78	4781.80±47.68	0.152

### B. Dosimetric comparison of normal tissue

The prone position plans significantly lower the exposure doses to the LAD, LV, heart, and ipsilateral lung (Fig. 3 and Table 2). The LAD‐PRV, LV, heart, and L‐Lung received smaller doses in the prone position plans than in the supine position plans. The Dmean values reduced by 445.83cGy(p=0.043),575.00cGy(p=0.003),402.00cGy(p=0.039), and 553.33cGy(p=0.004) in the LAD‐PRV, LV, heart, and L‐Lung. In addition, the V25 lessened 12.54%(p=0.042) and 8.70%(p=0.019) in the LV and heart, while the V20 was decreased 8.57%(p=0.042),15.21%(p=0.026),12.59%(p=0.011), and 10.62%(p=0.006) in the LAD‐PRV, LV, heart, and L‐Lung. Similarly, the V10 and V30 were reduced by 28.31%(p=0.029) and 5.54%(p=0.034) in the heart, while the V5 was cut back27.86%(p=0.031) in the L‐Lung (Table 2).

**Table 2:** Mean dose and volume indicators of normal tissue (n=10) in the prone and supine positions

*OAR*	*Dose/Volume*	*Prone Plan*	*Supine Plan*	*p‐value*
LAD‐PRV	Dmean(cGy)	2626.50±675.70	3072.33±716.09	0.043
	V10(%)^a^	77.15±18.10	91.73±8.37	0.064
	V20(%)	66.41±21.78	74.98±21.49	0.042
	V25(%)	53.11±22.65	65.43±24.58	0.105
	V30(%)	40.19±20.45	55.13±25.28	0.146
	V40(%)	22.00±12.85	31.40±16.26	0.259
LV	Dmean(cGy)	1176.33±452.21	1751.33±577.76	0.003
	V10(%)	48.54±27.53	81.14±16.60	0.005
	V20(%)	14.84±13.88	30.05±23.58	0.026
	V25(%)	8.72±8.34	21.26±18.35	0.042
	V30(%)	5.25±4.20	13.94±14.82	0.146
	V40(%)	0.94±1.59	3.71±5.95	0.192
Heart	Dmean(cGy)	1134.83±482.13	1536.83±492.79	0.039
	V10(%)	43.03±26.68	71.34±24.75	0.029
	V20(%)	13.75±8.16	26.34±13.11	0.011
	V25(%)	7.89±4.90	16.59±9.68	0.019
	V30(%)	4.02±2.60	9.56±5.77	0.034
	V40(%)	1.29±1.06	2.49±2.33	0.228
L‐Lung	Dmean(cGy)	861.17±320.97	1414.50±222.43	0.004
	V05(%)	57.79±27.80	85.65±9.41	0.031
	V20(%)	10.25±5.14	20.87±4.87	0.006
R‐Lung	Dmean(cGy)	258.00±106.28	439.20±201.18	0.070
	V05(%)	10.35±9.32	37.97±26.31	0.083
R‐Breast	Dmax(cGy)	591.20±117.15	809.60±184.40	0.124
	Dmean(cGy)	218.00±57.87	258.40±25.64	0.164

a 8

## IV. DISCUSSION

Local regional control and overall survival have been improved in patients receiving whole breast radiotherapy after breast‐conserving surgeries. However, the non‐breast cancer related mortality of these patients 15 years postradiotherapy has also increased unexpectedly.^(8)^ The long‐term cardiac toxicity has long been thought an important issue in breast cancer radiotherapy. Many studies have reported that radiation‐related late cardiovascular damage was attributed to the increased non‐breast cancer related death rate and was a trade‐off for the increased local control and overall survival. Wang et al.^(24)^ tracked 12,696 patients with breast cancer from 1995 to 2005 and found that coronary artery dysfunction was not more increased in left breast cancer patients than in right ones at an early time after radiotherapy; however, the risk significantly increased in these patients after 10–15 years. They also found that artery stenosis was the major abnormality according to CTA. A recent study on the dose‐volume effect of the heart showed that cardiovascular‐related deaths 15 years postradiotherapy would be less than 1% if the V25 of the heart was less than 10%. The LV and LAD likely received higher doses than the other substructures of the heart.^4^, ^5^ Taylor et al.^(2)^ also reported that in their studies, the LAD region received the highest dose among the heart substructures.

The purpose of this study was to evaluate the feasibility of using 64‐slice spiral CTA to help delineate the substructures of the heart in patients that underwent left breast‐conserving surgery, and to compare the dosimetric difference between IMRT plans in the prone and supine positions. In most studies, the LAD was contoured based on the anatomy instead of based on an image of the virtual structure,^16^, ^19^ which may have caused increased intra‐observer bias. The LAD can also be observed during PET scanning. PET‐CT was also used to help delineate the LAD.^(20)^ However, the high expense and long scanning time prevents its widespread clinical use. In this study, CTA was evaluated for the purpose of delineating the LAD. Coronary angiography displayed the LAD, LV, and cardiac substructures clearly, and at a lower cost than with PET‐CT. No patients complained of discomfort during the procedure. Because the structures were viewable, the intra‐observer bias was minimized.

To our knowledge, there are few studies on breast cancer radiotherapy in which patients were treated in the prone position and used CTV technology to contour the LAD. In the current study, these two approaches were combined, which provided details on the dose distribution difference between the supine and prone positions. Firstly, we found that there was no significant difference in dose uniformity of the target volume between the prone and supine positions, which was consistent with other studies.^13^, ^19^ However, in terms of critical organ protection, especially saving heart function, the prone position was superior to the supine position. With images provided by the CTA series, the substructure of the heart was precisely delineated in the current study and a dose‐volume histogram was generated. The results clearly showed that in the prone position the LAD, LV, and ipsilateral lung received a significantly lower dose than in the supine position. For most Asian women with average‐sized breasts, the prone position is a better choice for postoperation radiotherapy. CTA‐assisted CT scanning is recommended to help delineate the LAD and LV. Dose constraints for these critical structures should be applied in IMRT planning to reduce their exposed dose and to minimize late cardiac toxicities in these patients.

One of the limitations of the current study is that breath holding and other motion management were not applied during the simulation; thus, the planning risk volumes (PRV) dose may be not equal to the actual dose. Kirby et al.^(15)^ and Ramella et al.^(25)^ stratified patients based on breast size in their studies, which suggested that the size of the breast is a factor that needs to be taken into consideration. Our next study will include a relatively large number of patients, which will facilitate the subgrouping of patients with different breast sizes. Respiratory gating technology will be considered in order to obtain the precise dose of the organ of interest. The use of dose constraints for the LAD and LV is expected to help reduce the cardiac toxicities of breast cancer radiotherapy. Patients enrolled in the current and future studies will be carefully followed up, and the heart function of each patient will be evaluated. We expect that dose constraints for the LAD and LV in IMRT planning will be established by analyzing the correlation between the LAD and LV dose and late cardiac toxicities. In addition, a disadvantage of IMRT is the associated increased cost.

## V. CONCLUSIONS

CTA proved to be a simple, straightforward, and objective way to contour the substructure of the heart. Treatment planning with LAD and LV delineation indicated that a prone position is superior to a supine position for postoperation breast cancer radiotherapy. The exposure doses for the LAD, LV, heart, and ipsilateral lung in the prone position were all lower than in the supine position, although both techniques met the dose distribution requirement for the treatment volume. These conclusions will be further evaluated in a future study with a larger sample size, and dose constraints for the critical substructures of the heart are expected to be derived after a long‐term follow‐up.
